# Knowledge and practices of dairy farmers relating to brucellosis in urban, peri-urban and rural areas of Assam and Bihar, India

**DOI:** 10.1080/20008686.2020.1769531

**Published:** 2020-05-31

**Authors:** Ram Pratim Deka, Ulf Magnusson, Delia Grace, Rajeswari Shome, Johanna F. Lindahl

**Affiliations:** a Department of Clinical Sciences, Swedish University of Agricultural Sciences, Uppsala, Sweden; b Department of Animal & Human Health, International Livestock Research Institute(ILRI), Nairobi, Kenya; c Food safety System, Natural Resources Institute,University of Greenwich, UK; d ICAR- National Institute of Veterinary Epidemiology & Disease Informatics, Bengaluru, India; e Department of Medical Biochemistry and Microbiology, Uppsala University, Uppsala, Sweden

**Keywords:** Brucellosis, zoonotic disease, dairy production, India, farmers’ awareness

## Abstract

**Background:**

Brucellosis is one of the most common zoonotic diseases in the world. This study aimed at assessing farmers’ knowledge about brucellosis as well as practices relevant to transmission of brucellosis and their associated determinants.

**Results:**

Few farmers knew about brucellosis (3.4%, *n* = 18) and its zoonotic importance (0.8%, *n* = 4). Knowledge about brucellosis was higher for farmers with a larger herd size (*p* < 0.001) and fully using a stall-fed system (*p* < 0.001). Training on dairy cattle management (*p* < 0.001), training on animal disease (*p* < 0.01), consultation with veterinarians (*p* < 0.001) and farms being in urban areas (*p* < 0.01) were also significantly positively associated with knowledge about brucellosis. No significant association was observed between farmers’ knowledge about brucellosis and state, family size, education, age or gender of the farmers. Farmers knowledge about brucellosis was significantly associated with certain practices that include use of disinfectant while cleaning farms (*p* < 0.05), animal movement (*p* < 0.01), introduction of new animals (*p* < 0.05) and raw milk consumption (*p* < 0.05). The study did not find any association between knowledge about brucellosis and method of disposal of aborted materials, personal hygiene and quarantine practices.

**Conclusion:**

More interaction with veterinarians and training on animal management may be an important tool for generating awareness among the farming community for reducing transmission of the disease.

## Introduction

Brucellosis is one of the most important zoonotic diseases in the world [1,2]. In sexually mature female cattle, the disease causes abortion, typically in the last trimester, resulting in production losses [3]. Because of its zoonotic nature, brucellosis can spread to humans from animals through consumption of raw milk or milk products and through direct or indirect contact with aborted materials [4].

Brucellosis is endemic in parts of Africa, Central and South America, Middle East and Asia in both humans and animals [1]. In India, the disease is endemic [5], and *Brucella* sero-prevalence in bovines is reported as around 12%, but highly varying between studies [6]. Disease in humans is reported sporadically in India [7]. Thakur et al. [8] reported 5% sero-prevalence among 352 people professionally exposed to animals, Sen et al. [9] found 6.8% sero-positive cases among the patients with pyrexia of unknown origin and Shome et al. [10] recorded overall prevalence of 7.04% in personnel engaged in veterinary health care in Karnataka, India.

Prevalence of brucellosis in both humans and animals appears to be increasing because of dearth of awareness, policies and resources [1]. A study from India suggests that poor knowledge of brucellosis is significantly associated with prevalence of the disease [11], arguing a vicious cycle between underreporting/ under diagnosis and lesser awareness of farmers [12]. Therefore, assessment of knowledge and practices among the farming community is important for designing a disease knowledge dissemination program. In this study, we have assessed farmers’ knowledge about brucellosis and practices relevant for brucellosis transmission in urban, peri-urban and rural areas and their association with several determinates.

## Materials and methods

### Data and sampling procedure

The cross-sectional study was conducted in two Indian states, Assam and Bihar, through a primary survey of 534 dairy farming households during 2015–2016. Both the states are among the poorest in India (Assam ranked at 30 and Bihar at 33 out of 33 states in India) and located in Eastern/ North Eastern India where dairy industry is lagging behind than the rest of the country. In addition, a high *Brucella* sero-prevalence has been reported in both the states but there is little research on the subject. Lack of information on the subject may hinder appropriate preventive measures for reducing transmission of brucellosis from animals to humans and therefore both the states were considered for conducting the study. Since prevalence of brucellosis and their possible determinants may vary in urban, peri-urban and rural areas, therefore we conducted the study in three different settings. Here urban areas mean the areas which are under the administrative division of town committee/municipal corporation/council (local administrative body of urban areas), rural areas mean the areas that are under village *panchayat* (local village level administrative body) and the peri-urban areas that are under village panchayat but adjoining to urban areas.

Assuming 15% household level sero-prevalence, 95% level of confidence and 5% precision in the estimates, we needed at least 196 sample observations [13], and to account for an assumed small design effect, because of clustering, we aimed at 240 households in each state. Here household means family members who normally live together and take food from a common kitchen [14].

Multistage sampling technique was used to select the households in both the states. In the first stage, three districts were purposefully selected in each state to represent different distric potential for dairy development (low, medium and high). Availability of primary laboratory support and safety of the study team were also considered during the selection of the districts. In the second stage, two community development blocks (CDBs) from each district (one rural and one urban) were randomly selected. In Patna district, one peri-urban CDB was also selected because of the vibrant peri-urban dairy system near Patna city. In the third stage, four villages were selected randomly from the list of villages in each CDB. In the fourth stage, 10 households were selected randomly from the list of households having large ruminants (cattle and buffalo) from each selected village. Random selections were done by assigning computer generated random numbers. The primary survey was carried out by a team led by the first author, and locally recruited enumerators well versed with the local language and trained before conducting the study. The survey was conducted using a structured questionnaire with questions related to farmers, farming systems and their prevailing knowledge and practices towards disease management with a focus on brucellosis. Knowledge about brucellosis was assessed by asking the farmers if they had heard the disease called ‘brucellosis’ (there is no local term for the disease, the English word ‘brucellosis’ is the term used by veterinarians). The questionnaire was pre-tested during a pilot survey in each state. Slight modifications were made after completing the survey in Bihar and before starting in Assam, including adding some questions. The member of the farming family responsible for management of dairy animals was interviewed in local language. There was also an observation checklist, which was filled by the interviewer during the visit. At the beginning of the interview farmers were informed about the purpose of the study and their consent were taken using a customized consent form.

### Data analyses

Data were entered in Excel and analyzed using Stata 14 (STATA Corp. Ltd). The division of urban, peri-urban and rural was based on the official classification of the CDBs. The classification of farms into small (1–3 dairy animals), medium (4 − 10 dairy animals) and large farms (more than 10 dairy animals) was made according to the classification made by an FAO report [15].

A correlation matrix for different independent variables was constructed (Table 1). Unconditional associations between two binary or categorical variables (nominal or ordinal) were assessed by the Chi2 test. For multivariable analysis, multilevel mixed effects logistic regression model was used including the random effect of districts, blocks and villages, while states were included as fixed effects. Initially, all the possible variables were included as independent variables in the model and insignificant variables dropped one after another based on level of insignificance. In the final model, only the significant variables (*p* < 0.05) were included. Potential confounders were not kept in the model, and were not tested for.

**Table 1.** Correlation matrix among different independent variables.

Variables	Location	Gender	Age	Education	Farm category	Rearing system	Training on management	Training on disease	Vaccination	Disinfection	Veterinary consultation	Animal movement	Animal introduced	Consumption of raw milk
Location of farms	1.00													
Gender	−0.04	1.00												
Age	−0.03	0.03	1.00											
Education	0.07	0.07	0.12*	1.00										
Farm category	0.23*	0.10*	0.17*	0.15*	1.00									
Rearing system	0.30*	0.12	0.15*	0.13*	0.70*	1.00								
Training on management	0.16*	0.07	0.08	0.03	0.34*	0.27*	1.00							
Training on disease	0.09	0.05	0.11	0.07	0.14*	0.10	0.67	1.00						
vaccination	0.33	0.04	0.07	0.05	0.62*	0.62*	0.28	0.14	1.00					
Disinfection	0.07*	0.11*	0.00	0.02*	0.31*	0.41*	0.08	0.01	0.27*	1.00				
Veterinary consultation	0.28*	0.03	0.04	0.109	0.19*	0.30*	0.11	0.05	0.28*	0.20*	1.00			
Animal movement	−0.29*	−0.13	−0.17*	−0.13*	−0.70*	−0.98*	−0.28	−0.10	−0.62*	−0.41*	−0.29*	1.00		
Introduction of animals	0.12	0.09	−0.10	−0.17	0.48	0.41	0.22	0.10	0.33	0.28*	0.04	−0.42*	1.00	
Consumption of raw milk	0.15	0.03*	−0.04	−0.06*	0.15	0.23	0.05	0.09	0.23*	0.13*	0.08	−0.24	0.02	1.00

*p < 0.05

The study design and all tools used for the study were approved by Institutional Research Ethics Committee (IREC) at International Livestock Research Institute (ILRI) ILRI-IREC 2015–12 and IREC 2017–01, IACUC 2017–05.

## Results

### Description of sample households

Out of the total interviewed households (*n* = 534), 46.3% (*n* = 247) were from rural areas and another 46.3% (*n* = 247) were from urban areas. The remaining 7.4% (*n* = 40) households were from peri-urban areas in Bihar. Mean household size in Assam (5.6) was lower than in Bihar (7.8). Characteristics of the respondents in terms of farm size, age, gender, level of education and rearing system are presented at Table 2. In Assam, we found that few farmers (2.5%, *n* = 6) obtained training in rural areas compared to farmers in urban areas (7.0%, *n* = 17) which was statistically significant (*p* < 0.05). In addition, we found that significantly (*p* < 0.001) more large farms (78.0%) in Assam were in urban areas and more large-scale farmers (32.5%) (*p* < 0.001) had received training than medium or small-scale farmers (4.9%). Similarly, significantly (*p* < 0.001) more urban farmers (88.43%) had consulted veterinarians than rural farmers (64.46%) and more urban farmers (65.8%) reared dairy animals under fully stallfed system than rural farmers (51.4%).

**Table 2.** Farms and farmers’ characteristics with level of significance (*p* < 0.05) between Bihar and Assam states.

Variable	Bihar	Assam	Total	*p*-value
Small farms (1–3 dairy animals)	78.8% (*n* = 230)	76.5% (*n* = 185)	77.7% (*n* = 415)	0.009
Medium farms (4–10 dairy animals)	18.2% (*n* = 53)	14.5% (*n* = 35)	16.5% (*n* = 88)	
Large farms (>10 dairy animals)	3.1% (*n* = 9)	9.1% (*n* = 22)	5.8% (*n* = 31)	
Age of farmer (40 years or below)	41.1%(*n* = 120)	21.9%(*n* = 53)	32.4%(*n* = 173)	<0.001
Age of farmer (41 years or above)	58.9%(*n* = 172)	78.1%(*n* = 189)	67.6% (*n* = 361)	
Gender of farmers (male)	89.72%(*n* = 262)	95.87%(*n* = 232)	92.51%(*n* = 494)	0.007
Gender of farmers (female)	10.27%(*n* = 30)	4.13%(*n* = 10)	7.49%(*n* = 40)	
Education (below class 10)	57.9%(*n* = 169)	70.2%(*n* = 170)	63.5%(*n* = 339)	0.003
Education (above class 10)	42.1%(*n* = 123)	29.8%(*n* = 72)	36.5%(*n* = 195)	
Fully stall-fed rearing (zero-grazing)	85.3%(*n* = 249)	27.7%(*n* = 67)	59.2%(*n* = 316)	<0.001
Partly stall-fed rearing	14.7%(*n* = 43)	72.3%(*n* = 175)	40.8%(*n* = 218)	
Artificial insemination	91.1%(*n* = 266)	52.5%(*n* = 127)	73.6%(*n* = 393)	<0.001

### Farmers’ knowledge about brucellosis

Knowledge of farmers about brucellosis was assessed through a set of questions. It was observed that only 3.4% farmers in both the states reported that they knew something about brucellosis, another 4.7% farmers had heard the term brucellosis but did not know anything about it, and the remaining 91.9% farmers had not even heard about brucellosis. We did not observe any significant difference between Bihar and Assam in respect of knowledge about brucellosis (Table 3). For further analysis, we considered all the farmers who knew something about brucellosis and those who heard the term brucellosis as one group called who knew about brucellosis.

**Table 3.** Frequency distribution of farmers’ knowing about brucellosis with level of significance (*p* < 0.05) between Bihar and Assam states.

Knowledge on brucellosis	Bihar	Assam	Total	*p*-value
Know what brucellosis is	2.4%(*n* = 7)	4.6%(*n* = 11)	3.4%(*n* = 18)	0.30
Heard the name brucellosis but don’t know what it is	4.1%(*n* = 12)	5.4%(*n* = 13)	4.7%(*n* = 25)	
Don’t know anything about brucellosis	93.5% (*n* = 273)	90.1% (*n* = 218)	91.9% (*n* = 491)	

The farmers who reported that they knew about brucellosis were asked some additional questions related to brucellosis with multiple options. The results suggest that most common knowledge among farmers was that brucellosis affects cattle and it causes abortion in pregnant animals. Less than 1.0% of the farmers knew that brucellosis could transmit from animals to humans. It was observed that the level of knowledge in regards to brucellosis was almost the same between both the states (Table 4).

**Table 4.** Farmers’ knowledge about brucellosis between Bihar and Assam.

Knowledge on brucellosis	Bihar	Assam	Total
Farmers who knew something about brucellosis	2.4%(*n* = 7)	4.6%(*n* = 11)	3.4%(*n* = 18)
Farmers who knew *Brucella* affects cattle	2.4%(*n* = 7)	4.6%(*n* = 11)	3.4%(*n* = 18)
Farmers who knew *Brucella* affects buffalo	1.4%(*n* = 4)	0	0.8%(*n* = 4)
Farmers who knew *Brucella* affects human	1.0%(*n* = 3)	0.4%(*n* = 1)	0.8%(*n* = 4)
Farmers who knew human symptoms	1.4%(*n* = 4)	0	0.8%(*n* = 4)
Farmers who knew animal symptoms	2.4%(*n* = 7)	3.7% (*n* = 9)	3.0%(*n* = 16)
Believe *Brucella* vaccine available	1.0%(*n* = 3)	1.7%(*n* = 4)	1.3%(*n* = 7)
Know *Brucella* can transmit animals to human	0.3%(*n* = 1)	1.2%(*n* = 3)	0.8%(*n* = 4)
Know *Brucella* can transmit animal to animal	1.0% (*n* = 3)	0	0.6%(*n* = 3)
Know *Brucella* transmit through milk	1.4%(*n* = 4)	1.7%(*n* = 4)	1.5%(*n* = 8)
*Brucella* been diagnosed in the farm	0	0.8%(*n* = 2)	0.4%(*n* = 2)
Symptoms in human as mentioned	Prolonged fever (*n* = 3)		
Symptoms in animals as mentioned	Abortion (*n* = 12), vaginal discharge (*n* = 1), reduced milk (*n* = 1)		

### Determinants associated with farmers’ knowledge about brucellosis

Using univariable analyses (Chi2) between knowledge about brucellosis and different possible determinants that may associate with it we found that significantly (*p* < 0.01) more urban farmers (11.7%) knew about brucellosis than peri-urban (2.5%) or rural farmers (5.3%). Further, more farmers (*p* < 0.001) having large (38.7%) or medium (14.8%) sized farms knew about brucellosis than small sized farms (4.5%). Knowledge about brucellosis was also higher (*p* < 0.001) among farmers who reared dairy animals under fully stall-fed system (zero-grazing) (11.7%) than partly stall-fed system (2.8%). Besides, more farmers (*p* < 0.001) who had training on farm management (34.8%), training on diseases management (30.8%) and consultation with veterinarians (at least once in the year of survey) (12.9%) knew about brucellosis. However, no significant association was observed between farmers’ knowledge about brucellosis and state (*p* = 0.15), family size (*p* = 0.51), education (*p* = 0.22), age (*p* = 0.72) and gender (*p* = 0.18) of the farmers.

Using multivariable analysis, it was found that brucellosis knowledge was significantly associated with medium (*p* < 0.05) and large sized farms (*p* < 0.01) comparing with small sized farms (Table 5) (odds 2.4 and 6.7 times higher, respectively). Fully stall-fed system of rearing was also significantly (*p* < 0.05) associated with the knowledge about brucellosis (odds 2.9 times higher in case of fully stall-fed rearing).

**Table 5.** Final multivariable analysis of determinants having association with farmers’ knowledge about brucellosis.

Variable name	Odds ratio	95% confidence interval	p-value
Medium size (4–10 dairy animals)* farms compared to small size (1–3 dairy animals)* farms	2.4	0.99–5.69	0.05
Large size (>10 dairy animals)* farms compared to small size (1–3 dairy animals)	6.7	2.18–20.33	<0.001
Fully stall-fed system compared to partly stall-fed	2.9	1.03–7.88	0.04

### Farmers’ practices relevant for transmission of brucellosis

The univariable analysis indicated that knowledge about brucellosis was significantly associated with a few practices that are relevant for brucellosis transmission. The practices include consumption of raw milk (*p* < 0.05), use of disinfectant in cleaning the farm (*p* < 0.001), animal movement (*p* < 0.001) and introduction of new animal (*p* < 0.05). However, we did not find any significant association between farmers’ knowledge about brucellosis and practice of cleaning udder before milking (*p* = 0.59), throwing away placenta outside the farms (*p* = 0.16), burying the placenta (*p* = 0.26), washing of hands after handling aborted materials (*p* = 0.86), taking bath after handling aborted materials (*p* = 0.77), introduction of new animal (*p* = 0.11) and quarantine practice followed (*p* = 0.61). Only one farmer reported to use protective clothing like gloves while handling aborted materials.

Practices having significant association with knowledge about brucellosis were further studied by using multilevel mixed effects logistic regression model to see their association with some of the farms/farmers’ characteristics. The results are shown in Table 6.

**Table 6.** Odds ratio (95% confidence interval) of predictors for farmers’ practices relevant for transmission of brucellosis.

Independent factors	Response type	Consumption of raw milk	Use of disinfectant	Movement of animals	Introduction of new animal
Knowledge about brucellosis	Know Don’t know	3.3 (1.2–8.6)* Reference	2.4(1.2–5.0)* Reference	NS	NS
State	Assam Bihar	0.08(0.02–0.3)*** Reference	NS	NS	0.2(0.1–0.3)*** Reference
Location of farms	Urban Rural	NS	NS	0.3(0.2–0.8)*** Reference	NS
Category of farms	Small (1–3 dairy animals) Medium (4–10 dairy animals) Large (>10 dairy animals)	NS	NS	NS	Reference 6.2(0.1–0.3)*** 18.8(7.1–49.0)***
Rearing system of dairy animals	Fully stall-fed Partly stall-fed	NS	2.6(1.6–4.2)*** Reference	0.01(0.0–0.03)*** Reference	NS

*p < 0.05, ** *p* < 0.01, and *** *p* < 0.001, NS: not significant.

It was found that significantly more farmers who knew about brucellosis consumed raw milk than those who did not know (odds 3.3 times higher in case of those who knew about brucellosis) (Table 6). Besides, significantly more farming households in Bihar (15.1%) consumed raw milk than in Assam (2.1%).

We observed that 39.4% farmers in Bihar and 36.1% farmers in Assam used disinfectants while cleaning the farms. Out of the total farmers who used disinfectant, only a small percentage of farmers (2.0%) in Assam used disinfectant daily, the remaining used it seldom. Significantly more farmers who knew about brucellosis (65.1%) used disinfectant than who did not know about the disease (35.64%). It was also found that significantly more dairy farmers who reared dairy animals under fully stall-fed system (46.8%) used disinfectant than those who reared under partly stall-fed system (25.2%).

More farmers in Assam moved their animals (43.1%) than Bihar (12.0%) for grazing, selling etc. We found that significantly (Table 6) more rural farmers (49.4%) moved their animals than urban (35.6%) or peri-urban (5.0%) farmers as grazing is a common practice in rural areas, more particularly in Assam. In addition, significantly more farmers rearing dairy animals under partly stall-fed systems (89.5%) moved their animals than those who reared under fully stall- fed condition (5.4%).

Significantly (Table 6) more farmers in Bihar introduced new animals (35.6%) compared to Assam (14.9%) and more large sized farms (67.7%) and medium sized farms (54.5%) introduced new animals than small (17.1%) sized farms.

## Discussion

Our study finds that farmers’ knowledge about brucellosis in Bihar and Assam was very poor, similar to studies in the rest of India and neighboring countries. A study conducted in Puducherry, India reported that 4.8% farmers knew about brucellosis [15]. Similarly in Sri Lanka, only 2.6% farmers knew about brucellosis as a zoonotic disease [16]. Level of farmers’ knowledge widely varied among countries outside South Asia. For instance, in Senegal, none of the farmer knew about brucellosis [17]. On the contrary, in Tajikistan, 15.0% farmers [18]; in Ecuador, 30.0% farmers [19] and in Ethiopia, 48.0% of farmers [20] knew about brucellosis.

We found that only 2.4% farmers were aware that abortion occurs if animals suffer from brucellosis. This finding is in concurrence with a study from Sri Lanka which recorded only 8.3% farmers were aware about abortions due to brucellosis [17]. Relatively more farmers (19.2%) in South Africa were aware about abortions related to brucellosis [21]. In our study, only 0.8% farmers mentioned that brucellosis is transmitted from animals to humans. This finding is in agreement with a study from Pakistan where 3.0% of the farmers were aware of transmission of brucellosis from animals to human [22]. However, there have been reports from other countries with higher knowledge levels. In Portugal, 74.7% [23] and in Egypt, 96.3% farmers [24] were aware that brucellosis could be transmitted from animals to human.

We did not find any significant association between knowledge about brucellosis with farmers’ education, age and gender. Our study is in agreement with the finding of Diez and Coelho [23] who have reported that education, age and gender do not have any association with knowledge about brucellosis. A study in Tajikistan also did not find significant association between knowledge about brucellosis and gender [25]. Further, our finding is in contrast with the findings of Lindahl et al. [25] wherein farmers with lower level of education were less likely to have knowledge about brucellosis compared to highly educated people. Similar finding was reported from Pakistan [22], Kenya [26] and Ecuador [19].

In Assam, farmers’ knowledge about brucellosis had significant association with training availed by farmers and interaction with veterinarians. This finding is in agreement with a study finding from South Africa that the main source of knowledge among farmers about brucellosis was veterinary consultation [21]. Similarly, couple of other studies also reported that farmers consulting about animal health issues with veterinarians were more knowledgeable about brucellosis [23,25].

We observed that urban farmers were more aware of brucellosis. This might be because in urban areas accessibility to veterinary hospitals, doctors or other veterinary teaching, research and development organization was high. This relationship could also be partly explained by the fact that location of the farms had significant correlation with farm size, rearing system, training and consultation with veterinarians (Table 1). We did not find any significant association between farmers’ age and knowledge about brucellosis which is in contrast with the findings of Njuguna et al. [26] who found significant associations with both.

### Farmers’ practices relevant for transmission of brucellosis

A study from Mongolia reported brucellosis preventive practices were significantly associated with gender, location of farms, use of veterinary services and knowledge of brucellosis [27]. Our study is partly in agreement with this. Four farming practices, including consumption of raw milk, use of disinfectants in cleaning the farms, movement of animals and introduction of new animals are reported as important risk factors by some researchers [28–30] and we have found that these practices are significantly associated with either one or more farm/ farmers’ characteristics (viz. knowledge about brucellosis, location of the farms, rearing system and category of farms) (Table 6).

Consumption of raw milk is considered as an important risk factor for transmission of disease from animal to human [31,32]. Studies in Punjab and Sindh province of Pakistan found that about 66.0% of households consumed raw milk but only 3.0% were aware that brucellosis could be transmitted through milk [22]. The percentage of farmers consuming raw milk is even higher in some African countries like Senegal where 95% farmers reported to consume raw milk [18]. In our study, significantly more farmers who knew about brucellosis consumed raw milk, which was against our initial hypothesis. This might be because farmers had poor knowledge about transmission of brucellosis and therefore it failed to restrict consumption of raw milk. A study from Turkey reported that raw milk consumption had significant association with rural-urban consumers, age and economic condition [33]; however, we did not find such association.

It is reported that those farms who use disinfectant are at lower risk of being seropositive against *Brucella* [34]. Use of disinfectant in cleaning the farms was found significantly associated with knowledge about brucellosis and rearing system of dairy animals in our study. This might be because significantly more urban farmers reared dairy animals under fully stall-fed system and they might have used disinfectant in cleaning the farms as this category of farms produce more farm waste and nuisance. Besides, this group of urban farmers got more access to training and consultation with veterinarians and many of them might have knowledge about brucellosis.

A study from Sri Lanka reported that free movement of animals and introduction of new animals were both significantly associated with prevalence of brucellosis [35]. In our study, we found that significantly lesser number of farmers in urban areas moved their animals. This was possibly because more urban farmers reared dairy animals under fully stall-fed system because of scarcity of land for grazing or free movement.

Introduction of new animals was significantly associated with the category of farms and the states to which the farms belonged. This was possibly because medium and larger sized farms which were more common in Bihar introduced new animals to replace old/diseased/unproductive/less productive stock in order to keep the farms economically productive throughout the year.

While the study aimed at a completely random sampling of farms and animals, it is possible that the study was biased by incomplete sampling frames. In addition, responses of farmers may also be bias as farmers may report the practices that are desired but may not always follow, which may inflate the positive practices. However, given the lack of knowledge and lack of good practices reported, this is likely not a major bias in the paper.

## Conclusion

The study has shown that farmers’ knowledge about brucellosis in both Bihar and Assam states is negligible. Farmers’ knowledge about the disease is mainly associated with category of farms, rearing system, location of the farms, training availed on management/ disease and consultation with veterinarians. Some of these determinates again have correlation in-between. Further, knowledge of farmers about brucellosis might be useful in improving some of the farmers’ practices. The study indicated that the farmers’ knowledge about brucellosis might be increased if the farmers avail training and consult frequently with veterinarians. Therefore, a customized awareness programme may be designed to improve knowledge of farmers in order to increase adoption of brucellosis preventive practices. In doing so, important determinants identified in this study may be taken into consideration. However, more studies are required to have deeper understanding on the subject including on farmers’ attitude.
